# Antioxidant and Anticancer Constituents from the Leaves of *Liriodendron tulipifera*

**DOI:** 10.3390/molecules19044234

**Published:** 2014-04-03

**Authors:** Ya-Fei Kang, Chi-Ming Liu, Chiu-Li Kao, Chung-Yi Chen

**Affiliations:** 1 Department of Health Beauty, School of Medical and Health Sciences, Fooyin University, Ta-Liao District, Kaohsiung 83102, Taiwan; E-Mails: mt069@fy.edu.tw (Y.-F.K.); joe7day@yahoo.com.tw (C.-L.K.); 2 Tzu Hui Institute of Technology, Pingtung County 92641, Taiwan; E-Mail: beagleliu@gmail.com; 3 Department of Nutrition and Health Science, School of Medical and Health Sciences, Fooyin University, Ta-Liao District, Kaohsiung 83102, Taiwan

**Keywords:** *Liriodendron tulipifera*, scopoletin, antioxidative, (+)-norstephalagine

## Abstract

Sixteen compounds were extracted and purified from the leaves of *Liriodendron tulipifera*. These compounds include aporphines, oxoaporphine, coumarin, sesquiterpene lactone, benzenoids, cyclitol and steroids. (+)-Norstephalagine (**2**) (an aporphine) and scopoletin (**8**) (a coumarin) were isolated from *Liriodendron tulipifera* leaves from the first time. The identified compounds were screened for their antiradical scavenging, metal chelating and ferric reducing power activities. The results have showed that these compounds have antioxidative activity. The study has also examined the chemopreventive property of the isolated compounds against human melanoma cells A375. The results shown that (−)-anonaine (**1**), (−)-liridinine (**3**), (+)-lirinidine (**6**), lysicamine (**7**) and epitulipinolide diepoxide (**9**) significantly inhibited the proliferation of melanoma cells. These results revealed that these compounds have antioxidative activity and chemopreventive activity in skin melanoma cells.

## 1. Introduction

Free radicals and reactive oxygen species (ROS) are formed of hydrogen peroxide or superoxide anions [1]. Free radicals play an important role in different diseases [2]. The imbalance between the formation of ROS and the defenses provided by cell antioxidants will cause diseases, including cancer [3]. Skin cancer is the most prevalent cancer worldwide [4]. Skin cancer can be divided into melanoma and non-melanoma skin cancer depending on the cell type [4,5]. A lot of studies have demonstrated that diet and phytochemicals containing high level of antioxidants can decrease the incidence of cancers [6,7]. We have found a lot of phytochemicals from different plants with antioxidative activity and chemotherapeutic activity [8,9,10,11].

The genus *Liriodendron* (Magnoliaceae) contains two species, *L. tulipifera* and *L. chinense*. *L.* *tulipifera* is known as American tulip tree and is a hardwood native plant used for pulp and wood in furniture and paper-making in the United States. The bark of *L. tulipifera* was used by Native Americans as a febrifuge and for the treatment of fevers associated with malaria. Numerous phytochemical contituents including sesquiterpene lactones, alkaloids and sugar derivatives have been isolated from this species, however, few pharmacological studies of these phytochemicals are described. The antioxidant and anticancer activities of bark extracts of *L. tulipifera* were examined in previous studies [9,10].

**Figure 1:**
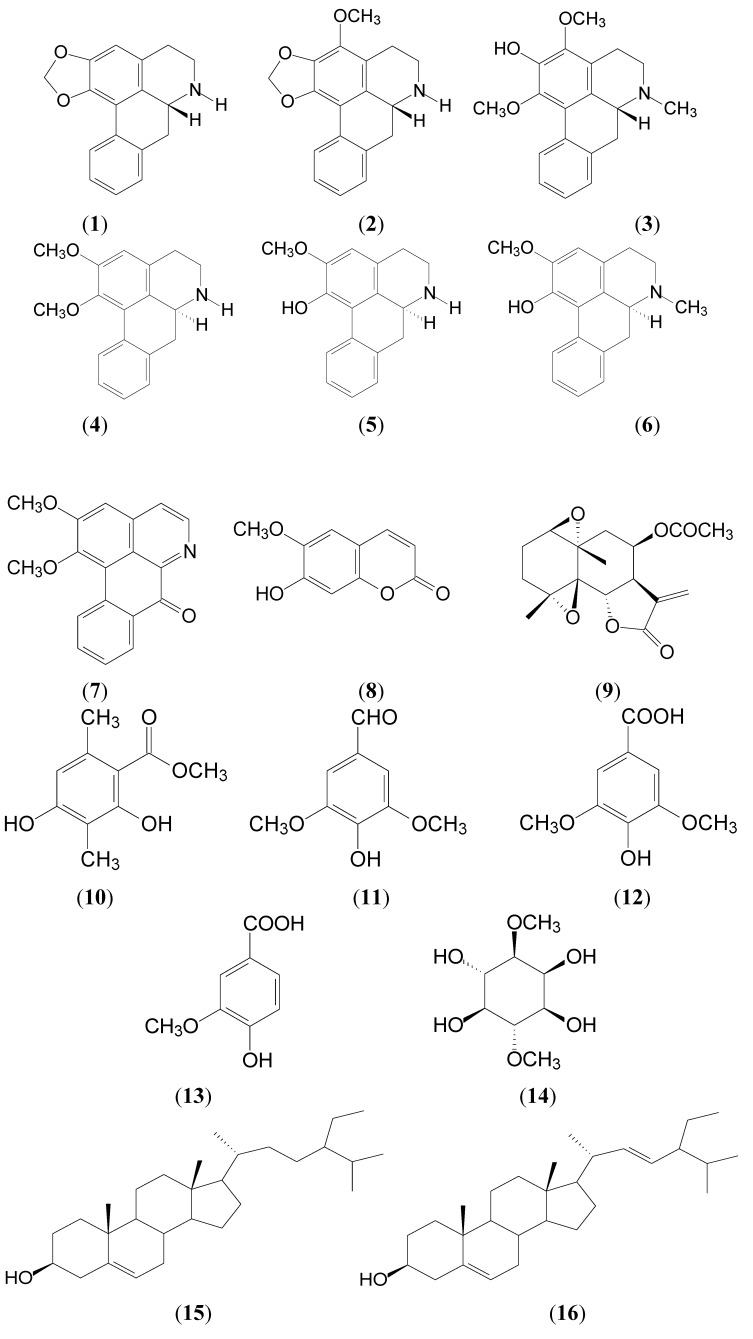
The chemical structures of compounds **1**–**16** from the leaves of *L. tulipifera*.

In the present study, we describe the isolation and characterization of several compounds from the leaves of *L. tulipifera*. These compounds are (−)-anonaine (**1**), (+)-norstephalagine (**2**), (−)-liridinine (**3**), (−)-nornuciferine (**4**), (+)-caaverine (**5**), (+)-lirinidine (**6**), lysicamine (**7**), scopoletin (**8**), epitulipinolide diepoxide (**9**), methyl β-orcinol carboxylate (**10**), syringaldehyde (**11**), syringic acid (**12**), vanillic acid (**13**), (−)-liriodendritol (**14**), β-sitosterol (**15**) and stigmasterol (**16**) whose structures are summarized in Figure 1. 

(+)-Norstephalagine (**2**) (an aporphine) and scopoletin (**8**) (a coumarin) were first isolated from the leaves of this species. The study also evaluates the antioxidant activity and anticancer activity of this species. To the best of our knowledge, this is the first study on investigating the antioxidant capacity and anticancer activities of scopoletin and (+)-norstephalagine from *L. tulipifera*.

## 2. Results and Discussion

### 2.1. Antioxidant Activities of Compounds **1** to **16** from L. tulipifera

The scavenging of radicals was measured in this study, as antioxidants act to inhibit the oxidation. The scavenging activities of compounds **1**–**16** at dosage of 100 μM were determined by a 1,1-diphenyl-2-picrylhydrazyl (DPPH) assay. As shown in Table 1, (+)-lirinidine (**6**) has minor radical scavenging activity (6.5%) compared with vitamin C (88.5%) at the same dose. The ferrous ion chelating activities are also shown in Table 1. EDTA (100 μM) was used as a positive control. Compounds **1**, **9**, **10**, **11**, **13** at the dosage of 100 μM displayed minor levels of Fe^2+^ scavenging effects of 2.9%, 1.8%, 4.6%, 1.4%, 3.4%, respectively. In the ferric reducing antioxidant power (FRAP) assay, the reducing power of the compounds **1**–**16** at 100 μM compared with 3-*tert*-butyl-4-hydroxyanisole (BHA) were shown in Table 1. (+)-Lirinidine (**6**) displayed the lowest activity, while compounds **1**–**3**, **5**, **7**–**11**, and **13**–**14** presented modest ferric reducing power. 

**Table 1:** Antioxidant activity of the extracted compounds at 100 μM. (-), no testing; (na), not active.

Compounds	DPPH (%)	Chelating (%)	Reducing power (OD 700)
Vitamin C a	88.5 ± 1.8	-	-
EDTA b	-	41.6 ± 4.5	-
BHA c	-	-	1.9 ± 0
(−)-Anonaine (**1**)	na	2.9 ± 0.0	0.1 ± 0
(−)-Norstephalagine (**2**)	na	na	0.1 ± 0
(−)-Liridinine (**3**)	0.5 ± 0	na	0.1 ± 0
(+)-Nornuciferine (**4**)	-	-	-
(+)-Caaverine (**5**)	na	na	0.2 ± 0
(+)-Lirinidine (**6**)	6.50 ± 0	na	0.7 ± 0
Lysicamine (**7**)	na	na	0.1 ± 0
Scopoletin (**8**)	na	na	0.4 ± 0
Epitulipinolide diepoxide (**9**)	na	1.8 ± 0	0.3 ± 0
Methyl β-orcinol carboxylate (**10**)	na	4.6 ± 0	0.2 ± 0
Syringaldehyde (**11**)	na	3.4 ± 0	0.3 ± 0
Syringic acid (**12**)	-	-	-
Vanillic acid (**13**)	na	3.39 ± 0	0.3 ± 0
(−)-Liriodendritol (**14**)	na	na	0.1 ± 0
β-Sitosterol (**15**)	-	-	-
Stigmasterol (**16**)	-	-	-

Data were expressed as a mean value of at least three independent experiments; ^a^ Vitamin C was used as a positive control on DPPH assay at 100 μM; ^b^ EDTA was used as a positive control on metal chelating ability at 100 μM; ^c^ BHA was used as a positive control on reducing power at 100 μM.

### 2.2. Anti-proliferation of compounds **1** to **16** in A375 cells

**Figure 2:**
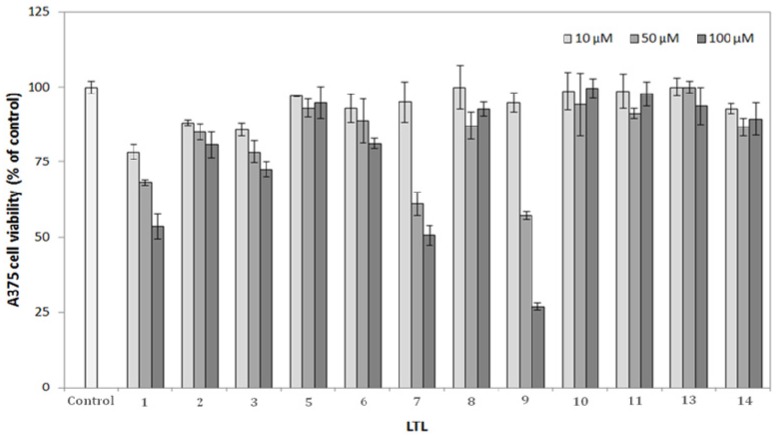
Anti-proliferative effects of *L. tulipifera* compounds on A375 cells. Cell growth was determined by MTT assay after incubation with 10, 50, 100 μM of compounds **1**–**14** respectively. Results are expressed as the percent of the cell proliferation of the vehicle control at 24 h.

The cytoxicity of compounds **1**-**16** was determined by an MTT assay. The melanoma cell line A375 cell line is metastatic and widely used in many studies. The A375 cells were treated with compounds **1** to **16** (1–100 μM) for 24 h. As shown in Figure 2, (−)-anonaine (**1**), (−)-liridinine (**3**), (+)-lirinidine (**6**), lysicamine (**7**) and epitulipinolide diepoxide (**9**) at 100 μM displayed sigificant cytoxicity in A375 cells. Particularly, the cell viability was less than 20% with epitulipinolide diepoxide (**9**) treatment at 24 h. The IC_50_ values of (−)-anonaine (**1**), lysicamine (**7**) and epitulipinolide diepoxide (**9**) were 97.16 μM, 58.12 μM and 52.03 μM. 

## 3. Experimental

### 3.1. General Procedures

UV spectra (MeCN) were measured on a Jasco UV-240 spectrophotometer (Jasco, Tokyo, Japan). IR spectra were measured on a Hitachi 260-30 spectrophotometer (Hitachi, Tokyo, Japan). ^1^H-NMR (400/500 MHz) and ^13^C-NMR (100 MHz), HSQC, HMBC, COSY and NOESY spectra were obtained on a Varian (Unity Plus) NMR spectrometer (Varian, San Francisco, CA, USA). For each sample, 128 scans were recorded with the following settings: 0.187 Hz/point; spectra width, 14400 Hz; pulse width, 4.0 μs; relaxation delay, 2 s. Low-resolution ESI-MS spectra were obtained on an API 3000 (Applied Biosystems, Foster City, CA, USA) and high-resolution ESI-MS spectra on a Bruker Daltonics APEX II 30e spectrometer (Bruker, Bremen, Germany). Silica gel 60 (Merck, 70~230 mesh, 230~400 mesh) was used for column chromatography. Precoated silica gel plates (Merck, Kieselgel 60 F-254), 0.20 mm and 0.50 mm, were used for analytical TLC and preparative TLC, respectively, and visualized with 10% H_2_SO_4_.

### 3.2. Plant Material

The specimen of *L. tulipifera* was collected from Chiayi County, Taiwan in December, 2007. A voucher specimen was characterized by Dr. Jin-Cherng Huang of Department of Forest Products Science and Furniture Engineering, National Chiayi University, Chiayi, Taiwan and deposited in the School of Medical and Health Sciences, Fooyin University, Kaohsiung, Taiwan.

### 3.3. Extraction, Isolation and Identification

The air-dried leaves of *L. tulipifera* (3.0 kg) were extracted with MeOH (50 L × 5) at room temperature and the MeOH extract (52.5 g) was obtained upon concentration under reduced pressure. This extract was chromatographed over silica gel using CH_2_Cl_2_/MeOH as eluent to produce five fractions. Part of fraction 2 (5.23 g) was subjected to silica gel column chromatography eluting with *n*-hexane/acetone (60:1) to furnish two fractions (2-1~2-2). Fraction 2-2 (2.47 g) was further purified on another silica gel column using *n*-hexane/acetone (10:1) to obtain methyl β-orcinol carboxylate (**10**, 6 mg). Part of fraction 3 (13.94 g) was subjected to silica gel column chromatography eluting with *n*-hexane/acetone (30:1), then enriched with acetone to furnish three fractions (3-1~3-3). Fraction 3-1 (3.81 g) eluted with *n*-hexane/EtOAc (25:1) was further separated using silica gel column chromatography and preparative TLC (*n*-hexane/EtOAc, 10:1) to give β-sitosterol (**15**) and stigmasterol (**16**). Fraction 3-2 (6.33 g) was further purified on a silica gel column using a CH_2_Cl_2_/MeOH eluent system to obtain syringaldehyde (**11**, 9 mg), (−)-liridinine (**3**, 12 mg) and scopoletin (**8**, 95 mg). Fraction 3-3 (2.43 g) eluted with *n*-hexane/acetone was further separated using silica gel column chromatography and preparative TLC (*n*-hexane/EtOAc, 4:1) to give lysicamine (**7**, 25 mg). Part of fraction 4 (10.45 g) was subjected to silica gel chromatography by eluting with CH_2_Cl_2_/MeOH (80:1), enriched with MeOH to furnish four fractions (4-1~4-4). Epitulipinolide diepoxide (**9**, 8 mg) was further purified on a silica gel column using a CH_2_Cl_2_/MeOH (40:1) system from fraction 4-2. Fraction 4-3 (3.27 g) eluted with CH_2_Cl_2_/MeOH (20:1) was further separated using silica gel column chromatography to obtain syringic acid (**12**, 7 mg) and vanillic acid (**13**, 12 mg). Fraction 4-4 (3.53 g) was further purified by another silica gel column using CH_2_Cl_2_/MeOH (10:1) to obtain (−)-anonaine (**1**, 10 mg). Part of fraction 5 (17.21 g) was subjected to silica gel column chromatography by eluting with CH_2_Cl_2_/MeOH (30:1), enriched with MeOH to furnish three fractions (5-1~5-3). Fraction 5-1 (4.12 g) eluted with CH_2_Cl_2_/MeOH (15:1) was further separated using silica gel column chromatography to give (+)-lirinidine (**6**, 10 mg) and (+)-norstephalagine (**2**, 12 mg). Fraction 5-2 (3.82 g) was further purified by silica gel column using CH_2_Cl_2_/MeOH (8:1) to obtain (−)-nornuciferine (**4**, 10 mg) and (+)-caaverine (**5**, 14 mg). Fraction 5-3 (8.97 g) was purified by recrystallization to obtain (−)-liriodendritol (**14**, 310 mg).

(−)*-Anonaine* (**1**)*.* Yellow needles (MeOH); UV λ_max_: 230, 272, 310 nm; IR ν_max_: 1040, 950 cm^−1^; ^1^H- NMR (500 MHz, CDCl_3_): δ 2.60 (1H, *d*, *J* = 12.0 Hz, H-7a), 2.85 (1H, *t*, *J* = 12.0 Hz, H-7b), 3.10~3.20 (3H, *m*, H-4a, 4b, 5a), 3.66 (1H, *m*, H-5b), 3.86 (1H, *m*, H-6a), 5.95 and 6.10 (each 1H, *d*, *J* = 1.0 Hz, -OCH_2_O-), 6.57 (1H, *s*, H-3), 7.21~7.34 (3H, *m*, H-8~10), 8.07 (1H, *d*, *J* = 7.5 Hz, H-11); ESI-MS *m/z*: 265 [M]^+^ [12].

(−)*-Norstephalagine* (**2**)*.* Brown powder (MeOH); UV λ_max_: 241, 280 nm; IR ν_max_: 1420, 1050, 950 cm^−1^; ^1^H-NMR (500 MHz, CDCl_3_): δ 2.18 (1H, *m*, H-7), 2.33~2.86 (4H, *m*, H-4, 5), 3.89 (1H, *m*, H-6), 3.93 (3H, *s*, C_3_-OCH_3_), 5.96 and 6.03 (each 1H, *d*, *J* = 1.8 Hz, -OCH_2_O-), 6.99~7.22 (3H, *m*, H-8~10), 8.16 (1H, *d*, *J* = 8.0 Hz, H-11); ESI-MS *m/z*: 295 [M]^+^ [13].

(−)*-Liridinine* (**3**)*.* Brown powder (MeOH); UV λ_max_: 221, 281 nm; IR ν_max_: 2830, 1595, 1290, 760 cm^−1^; ^1^H-NMR (500 MHz, CDCl_3_): δ 2.50 (3H, *s*, N-CH_3_), 3.65 (3H, *s*, C_1_-OCH_3_), 3.94 (3H, *s*, C_3_-OCH_3_), 6.97~7.24 (3H, *m*, H-8~10), 8.12 (1H, *d*, *J* = 8.0 Hz, H-11); ESI-MS *m/z*: 325 [M]^+^ [14].

(+)*-Nornuciferine* (**4**)*.* Brown powder (MeOH); UV λ_max_: 230, 272, 310 nm; IR ν_max_: 2900, 1590, 1440 cm^−1^; ^1^H-NMR (500 MHz, CDCl_3_): δ 2.81~3.11 (7H, *m*, H-4~7), 3.75 (3H, *s*, C_1_-OCH_3_), 3.93 (3H, *s*, C_2_-OCH_3_), 6.60 (1H, *s*, H-3), 7.16~7.29 (3H, *m*, H-8~10), 8.22 (1H, *d*, *J* = 8.0 Hz, H-11); ESI-MS *m/z*: 281 [M]^+^ [15].

(+)*-Caaverine* (**5**)*.* Brown powder (MeOH); UV λ_max_: 231, 271, 310 nm; IR ν_max_: 1760, 1625 cm^−1^; ^1^H- NMR (500 MHz, CDCl_3_): δ 3.14~3.78 (7H, *m*, H-4~7), 3.93 (3H, *s*, C_2_-OCH_3_), 6.60 (1H, *s*, H-3), 7.22~7.34 (3H, *m*, H-8~10), 8.37 (1H, *d*, *J* = 8.0 Hz, H-11); ESI-MS *m/z*: 267 [M]^+^ [15].

(+)*-Lirinidine* (**6**)*.* Deep green needles (CH_2_Cl_2_); UV λ_max_: 232, 272, 311 nm; IR ν_max_: 3400, 2850, 1630 cm^−1^; ^1^H-NMR (500 MHz, CDCl_3_): δ 2.58 (3H, *s*, N-CH_3_), 2.65~3.18 (7H, *m*, H-4~7), 3.92 (3H, *s*, C_2_-OCH_3_), 6.59 (1H, *s*, H-3), 7.21~7.33 (3H, *m*, H-8~10), 8.37 (1H, *d*, *J* = 8.0 Hz, H-11); ESI-MS *m/z*: 281 [M]^+^ [16].

*Lysicamine* (**7**)*.* Yellow needles (CH_2_Cl_2_); UV λ_max_: 255, 283, 335 nm; IR ν_max_: 3744, 1650, 1516 cm^−1^; ^1^H-NMR (500 MHz, CDCl_3_): δ 4.03 (3H, *s*, C_1_-OCH_3_), 4.12 (3H, *s*, C_2_-OCH_3_), 7.25 (1H, *s*, H-3), 7.58 (1H, *t*, *J* = 8.5 Hz, H-9), 7.78 (1H, *t*, *J* = 8.5 Hz, H-10), 7.84 (1H, *d*, *J* = 6.5 Hz, H-4), 8.58 (1H, *d*, *J* = 8.5 Hz, H-8), 8.96 (1H, *d*, *J* = 6.5 Hz, H-5), 9.19 (1H, *d*, *J* = 8.5 Hz, H-11); ESI-MS *m/z*: 291 [M]^+^ [12].

*Scopoletin* (**8**)*.* Yellow needles (CH_2_Cl_2_); UV λ_max_: 227, 261, 297, 344 nm; IR ν_max_: 3200, 1730 cm^−1^; ^1^H- NMR (500 MHz, CDCl_3_): δ 3.96 (3H, *s*, OCH_3_), 6.27 (1H, *d*, *J* = 9.5 Hz, H-3), 6.85 (1H, *s*, H-8), 6.92 (1H, *s*, H-5), 7.60 (1H, *d*, *J* = 9.5 Hz, H-4); ESI-MS *m/z*: 192 [M]^+^ [17].

*Epitulipinolide diepoxide* (**9**)*.* Yellow powder (CH_2_Cl_2_); UV λ_max_: 210 nm; IR ν_max_: 1770, 1745, 1660, 1245 cm^−1^; ^1^H-NMR (500 MHz, CDCl_3_): δ 1.38 (3H, *s*, H-14), 1.45 (3H, *s*, H-15), 2.09 (3H, *s*, OCH_3_), 3.08 (1H, *t*, *J* = 8.5 Hz, H-5), 4.48 (1H, *t*, *J* = 8.5 Hz, H-6), 5.70 (1H, *m*, H-8), 5.72 (1H, *d*, *J* = 3.0 Hz, H-13a), 6.41 (1H, *d*, *J* = 3.5 Hz, H-13b); ESI-MS *m/z*: 322 [M]^+^ [18].

*Methyl* β*-orcinol carboxylate* (**10**)*.* Colorless needles (CH_2_Cl_2_); UV λ_max_: 220, 236, 317 nm; IR ν_max_: 3400, 1690, 1590, 1515 cm^−1^; ^1^H-NMR (500 MHz, CDCl_3_): δ 2.11 (3H, *s*, C_3_-CH_3_), 2.47 (3H, *s*, C_6_-CH_3_), 3.93 (3H, *s*, COOCH_3_), 5.30 (1H, *s*, C_4_-OH), 6.21 (1H, *s*, H-5), 12.03 (1H, *s*, C_2_-OH); ESI-MS *m/z*: 196 [M]^+^ [19].

*Syringaldehyde* (**11**)*.* Colorless needles (MeOH); UV λ_max_: 216, 230, 308 nm; IR ν_max_: 3266, 1671 cm^−1^; ^1^H-NMR (500 MHz, CDCl_3_): δ 3.98 (6H, *s*, C_3_-OCH_3_ and C_5_-OCH_3_), 6.05 (1H, *s*, C_4_-OH), 7.16 (2H, *s*, H-2, 6), 9.83 (1H, *s*, C_1_-CHO); ESI-MS *m/z*: 182 [M]^+^ [13].

*Syringic acid* (**12**). Brown needles (CH_2_Cl_2_), UV λ_max_: 212, 235, 308 nm, IR ν_max_: 3255, 1670, 1514, 1330 cm^−1^; ^1^H-NMR (500 MHz, CDCl_3_): δ 3.89 (6H, *s*, C_3_-OCH_3_ and C_5_-OCH_3_), 7.33 (2H, *s*, H-2 and H-6); ESI-MS *m/z*: 198 [M]^+^ [13].

*Vanillic acid* (**13**). Colorless needles (ether), UV λ_max_: 220, 265, 300 nm, IR ν_max_: 3550, 1680, 1510, 1280 cm^−1^; ^1^H-NMR (500 MHz, CDCl_3_): δ 3.97 (3H, *s*, C_3_-OCH_3_), 6.98 (1H, *d*, *J* = 8.5 Hz, H-5), 7.60 (1H, *d*, *J* = 1.5 Hz, H-2), 7.73 (1H, *dd*, *J* = 8.5, 1.5 Hz, H-6); ESI-MS *m/z*: 168 [M]^+^ [13].

(−)*-Liriodendritol* (**14**). White needles (pyridine); IR ν_max_: 3300, 2900, 1500, 1370, 1100 cm^−1^; ^1^H- NMR (400 MHz, C_5_D_5_N): δ 3.41 (1H, *dd*, *J* = 9.6, 2.8 Hz, H-1), 3.55 (3H, *s*, C_1_-OCH_3_), 3.92 (3H, *s*, C_4_-OCH_3_), 4.01 (1H, *dd*, *J* = 9.6, 2.4 Hz, H-3), 4.04 (1H, *t*, *J* = 9.2 Hz, H-5), 4.17 (1H, *t*, *J* = 9.2 Hz, H-4), 4.69 (1H, *t*, *J* = 9.2 Hz, H-6), 4.72 (1H, *br s*, H-2), 5.03 (2H, *br s,* OH), 6.70 (2H, *br s*, OH); ESI-MS *m/z*: 208 [M]^+^ [20].

β*-Sitosterol* (**15**) & *Stigmasterol* (**16**). White needles (CH_2_Cl_2_); UV λ_max_: 205 nm; IR ν_max_: 3420, 2910, 1625, 1450 cm^−1^; ^1^H-NMR (500 MHz, CDCl_3_): δ 0.67 (3H, *s*, H-18 of **15**), 0.79 (3H, *d*, *J* = 7.0 Hz, H-26), 0.81 (3H, *d*, *J* = 7.0 Hz, H-27), 0.85 (3H, *d*, *J* = 7.5 Hz, H-29), 0.90 (3H, *d*, *J* = 7.5 Hz, H-29 of **15**), 0.99 (3H, *s*, H-19), 3.52 (1H, *m*, H-3), 5.03 (1H, *dd*, *J* = 15.2, 8.6 Hz, H-22 of **16**), 5.13 (1H, *dd*, *J* = 15.2, 8.6 Hz, H-23 of **16**), 5.36 (1H, *br s*, H-6); ESI-MS *m/z*: 414 [M]^+^, 412 [M]^+^ [12].

### 3.4. Determination of DPPH·Radical Scavenging Capacity

DPPH·is a stable free radial with a violet color (absorbance at 517 nm) that changes its color to light yellow when the free radicals are scavenged. Various concentrations of the four compounds were added to 0.1 mL of stable DPPH (60 μM) solution. When DPPH reacts with hydrogen-donating anti-oxidant, it is reduced, resulting in a decrease in absorbance at 517 nm. The analyzed time interval was 10 min per point, up to 30 min by using UV-vis spectrophotometer (Jasco, Tokyo, Japan). Vitamin C was acted as a positive control. The DPPH· radical scavenging activity (%) was determined as:

1 − [(*A*_control_ − *A*_sample_)/*A*_control_] × 100

### 3.5. Metal Chelating Activity

The ferrous ion chelating potential of the four *L. tulipifera* compounds was investigated according to a previously described method [9]. Briefly, various test concentrations of samples dissolved in DMSO were added to a solution of 2 mM FeCl_2_·4H_2_O (0.01 mL). The reaction was initiated by the addition of 5 mM ferrozine (0.02 mL), and the mixture was vigorously shaken and left standing at room temperature for 10 min. The absorbance of the mixture was then read at 562 nm against a blank. EDTA was used as a positive control. The metal chelating activity was determined as:

1 − [(*A*_control_ − *A*_sample_)/*A*_control_] × 100

### 3.6. Reducing Power

The reducing powers of our natural pure compounds were determined according to the method of [9]. Briefly, various concentrations of test samples were mixed with 67 mM phosphate buffer (pH 6.8, 0.085 mL) and 20% potassium ferricyanide [K_3_Fe(CN)_6_, 2.5 μL) The mixture was incubated at 50 °C for 20 min, and trichloroacetic acid (10%, 0.16 mL) was then added to the mixture that was then centrifuged for 10 min at 3000 g. The upper layer of the solution (75 μL) was mixed with 2% FeCl_3_ (25 μL), and the absorbance was measured with a 96-well plate spectrophotometer at 700 nm. A higher absorbance demonstrates a higher reductive capability.

### 3.7. Cell Culture

Human melanoma cell lines A375 were obtained from the American Type Cell Culture Collection (ATCC, Manassas, VA, USA). It was maintained in monolayer culture at 37 °C and 5% CO_2_ in DMEM supplemented with 10% FBS, 10 µg/mL of penicillin, 10 µg/mL of streptomycin and 0.25 μg/mL of amphotericin B.

### 3.8. Cell Viability Assay–MTT Assay

The MTT assay was used to determine cell viability and proliferation. The cell lines were seeded in 96-well culture plates (1 × 10^4^ cells/well). After seeding cells for 24 h, various compounds with concentration 100 μM were added. Within 24 h of compound treatments, images of human melanoma A375.S2 cells were taken at suitable time intervals. MTT solution (5 mg/mL and dissolved in phosphate buffered saline; PBS) was diluted 1:10 in culture medium and added to a culture dish followed by an incubation at 37 °C. After 2 h of MTT treatment, the media was removed and each precipitate in a specific dish was dissolved in 100 μL of DMSO to dissolve the purple formazan crystals. After the dishes were gently shaken for 20 min in the dark to ensure maximal dissolution of formazan crystals, the optical density (OD) values of the supernatant were measured at 595 nm. All experiments were repeated at least three times. In consideration of the possible anti-proliferative effects of DMSO, a maximal amount (0.5%) of DMSO was added to culture and used as positive controls. DMSO at this amount was found not to affect the growth of the human melanoma A375.S2 cells.

### 3.9. Statistical Analysis

All data are the means ± SD from at least triplicate experiments. The significance of the differences was analyzed by a one-way analysis of variance (ANOVA), with *p* < 0.05 or 0.01 as considered significant.

## 4. Conclusions

One study has shown that the bark and leaves extracts from *L. tulipifera* have antiplasmodial activity [21]. The leaf essential oil and methanolic extract of the leaves from *L. tulipifera* display anticancer activity [22,23]. From our current and previous study, we found that the leaf and bark extracts from *L. tulipifera* is abundant of antioxidants [9,10]. This study shows the antioxidant activity and anticancer activity of 16 constituents from the leaves of *L. tulipifera*. These constituents include aporphines, oxoaporphine, coumarin, sesquiterpene lactone, benznoids, cyclitol and steroids. (−)-Liriodendritol (**14**) is the most abundant component of the extract. (+)-Norstephalagine (**2**) (an aporphine) and scopoletin (**8**) (a coumarin) are isolated for the first time from *L. tulipifera*.

Among these compounds, (+)-lirinidine (**6**) displayed medium ferric reducing power activity and minor radical scavenging activity. A previous study has shown that lirinidine exhibited significant inhibition of collagen, arachidonic acid and platelet activating factor-induced platelet aggregation [24]. We suggest that (+)-lirinidine is a candidate for the cosmetic business and the food industry. Although scopoletin did not show potential anticancer and antioxidant activity in this study, many other studies have shown that scopoletin has anti-inflammatory and antioxidation activity *in vivo* and *in vitro* [25,26]. One study has shown that (+)-norstephalagine has relaxation activity on rat uterine smooth muscle. It is interesting that sesquiterpene lactone epitulipinolide diepoxide strongly inhibited melanoma cells (A375) with minor ferric reducing power activity. Moreover, one study has demonstrated that epitulipinolide diepoxide has cytotoxic activity against KB cells [27]. In the future, the mechanism of action of epitulipinolide diepoxide can be further examined in different cancer cells.

## References

[1] Wang X., Fang H., Huang Z., Shang W., Hou T., Cheng A., Cheng H. (2013). Imaging ROS signaling in cells and animals. J. Mol. Med..

[2] Dixon S.J., Stockwell B.R. (2014). The role of iron and reactive oxygen species in cell death. Nat. Chem. Biol..

[3] Saeidnia S., Abdollahi M. (2013). Antioxidants: Friends or foe in prevention or treatment of cancer: The debate of the century. Toxicol. Appl. Pharm..

[4] Rebecca V.W., Sondak V.K., Smalley K.S. (2012). A brief history of melanoma: From mummies to mutations. Melanoma Res..

[5] Lee C., Collichio F., Ollila D., Moschos S. (2013). Historical review of melanoma treatment and outcomes. Clin. Dermatol..

[6] Srinivasan K. (2014). Antioxidant potential of spices and their active constituents. Crit. Rev. Food Sci..

[7] Shehzad A., Lee J., Lee Y.S. (2013). Curcumin in various cancers. BioFactors.

[8] Chen B.H., Chang H.W., Huang H.M., Chong I.W., Chen J.S., Chen C.Y., Wang H.M. (2011). (−)-Anonaine induces DNA damage and inhibits growth and migration of human lung carcinoma h1299 cells. J. Agric. Food Chem..

[9] Chiu C.C., Chou H.L., Wu P.F., Chen H.L., Wang H.M., Chen C.Y. (2012). Bio-functional constituents from the stems of *Liriodendron tulipifera*. Molecules.

[10] Li W.J., Lin Y.C., Wu P.F., Wen Z.H., Liu P.L., Chen C.Y., Wang H.M. (2013). Biofunctional constituents from *Liriodendron tulipifera* with antioxidants and anti-melanogenic properties. Int. J. Mol. Sci..

[11] Chen C.Y., Cheng K.C., Chang A.Y., Lin Y.T., Hseu Y.C., Wang H.M. (2012). 10-Shogaol, an antioxidant from *Zingiber officinale* for skin cell proliferation and migration enhancer. Int. J. Mol. Sci..

[12] Chen C.Y., Chang F.R., Wu Y.C. (1997). The constituents from the stems of *Annona cherimola*. J. Chin. Chem. Soc..

[13] Chen C.Y., Chang F.R., Wu Y.C. (1999). Cheritamine, a new *N*-fatty acyl tryamine and other constituents from the stems of *Annona cherimola*. J. Chin. Chem. Soc..

[14] Abdusamatov A., Ziyaev R., Yunusov S. (1975). Alkaloids of *Liriodendron tulipifera*. Chem. Nat. Compd..

[15] Wang H.M., Yang W.L., Yang S.C., Chen C.Y. (2011). Chemical constituents from the leaves of *Nelumbo nucifera* Gaertn. cv. *Rosa-plena*. Chem. Nat. Compd..

[16] Ziyaev R., Arslanova O., Abdusamatov A., Yunusov S.Y. (1980). Alkaloids of *Liriodendron tulipifera*. Chem. Nat. Compd..

[17] Lee C.K., Lee P.H., Kuo Y.H. (2001). The chemical constituents from the aril of *Cassia fistula* L.. J. Chin. Chem. Soc..

[18] Doskotch R.W. (1975). New sesquiterpene lactones from *Liriodendron tulipifera*. Phytochemistry.

[19] Kouam S.F., Ngadjui B.T., Krohn K., Wafo P., Ajaz A. (2005). Prenylated anthronoid antioxidants from the stem bark of *Harungana madagascariensis*. Phytochemistry.

[20] Stadelmaier A., Schmidt R.R. (2003). Synthesis of phosphatidylinositol mannoside (PIMs). Carbohyd. Res..

[21] Graziose R., Rathinasabapathy T., Lategan C., Poulev A., Smith P.J., Grace M., Lila M.A., Raskin I. (2011). Antiplasmodial activity of aporphine alkaloids and sesquiterpene lactones from *Liriodendron tulipifera* L.. J. Ethnopharmacol..

[22] Miller S.L., Villanueva H.E., Palazzo M.C., Wright B.S., Setzer W.N. (2009). Seasonal variation and bioactivity in the leaf oil of *Liriodendron tulipifera* growing in Huntsville, Alabama. Nat. Prod. Commun..

[23] Moon M.K., Oh H.M., Kwon B.M., Baek N.I., Kim S.H., Kim J.S., Kim D.K. (2007). Farnesyl protein transferase and tumor cell growth inhibitory activities of lipiferolide isolated from *Liriodendron tulipifera*. Arch. Pharmacal Res..

[24] Chang F.R., Wei J.L., Teng C.M., Wu Y.C. (1998). Two new 7-dehydroaporphine alkaloids and antiplatelet action aporphines from the leaves of *Annona purpurea*. Phytochemistry.

[25] Dou Y., Tong B., Wei Z., Li Y., Xia Y., Dai Y. (2013). Scopoletin suppresses IL-6 production from fibroblast-like synoviocytes of adjuvant arthritis rats induced by IL-1beta stimulation. Int. Immunopharmacol..

[26] Witaicenis A., Seito L.N., da Silveira Chagas A., de Almeida L.D.J., Luchini A.C., Rodrigues-Orsi P., Cestari S.H., di Stasi L.C. (2014). Antioxidant and intestinal anti-inflammatory effects of plant-derived coumarin derivatives. Phytomedicine.

[27] Doskotch R.W., el-Feraly F.S. (1970). The structure of tulipinolide and epitulipinolide. Cytotoxic sesquiterpenes from *Liriodendron tulipifera* L.. J. Org. Chem..

